# Dietary Bioactive Ingredients Modulating the cAMP Signaling in Diabetes Treatment

**DOI:** 10.3390/nu13093038

**Published:** 2021-08-30

**Authors:** Yanan Wang, Qing Liu, Seong-Gook Kang, Kunlun Huang, Tao Tong

**Affiliations:** 1 Beijing Advanced Innovation Center for Food Nutrition and Human Health, College of Food Science and Nutritional Engineering, China Agricultural University, Beijing 100083, China; wangyanan_ytu@126.com; 2 Jilin Green Food Engineering Research Institute, Changchun 130022, China; jl_greenfood@163.com; 3 Department of Food Engineering, Mokpo National University, Muangun 58554, Korea; sgkang@mokpo.ac.kr; 4 Key Laboratory of Safety Assessment of Genetically Modified Organism (Food Safety), Ministry of Agriculture, Beijing 100083, China

**Keywords:** cAMP, diabetes, dietary bioactive ingredients, glucose homeostasis

## Abstract

As the prevalence of diabetes increases progressively, research to develop new therapeutic approaches and the search for more bioactive compounds are attracting more attention. Over the past decades, studies have suggested that cyclic adenosine monophosphate (cAMP), the important intracellular second messenger, is a key regulator of metabolism and glucose homeostasis in diverse physiopathological states in multiple organs including the pancreas, liver, gut, skeletal muscle, adipose tissues, brain, and kidney. The multiple characteristics of dietary compounds and their favorable influence on diabetes pathogenesis, as well as their intersections with the cAMP signaling pathway, indicate that these compounds have a beneficial effect on the regulation of glucose homeostasis. In this review, we outline the current understanding of the diverse functions of cAMP in different organs involved in glucose homeostasis and show that a diversity of bioactive ingredients from foods activate or inhibit cAMP signaling, resulting in the improvement of the diabetic pathophysiological process. It aims to highlight the diabetes-preventative or -therapeutic potential of dietary bioactive ingredients targeting cAMP signaling.

## 1. Introduction

**Figure 1:**
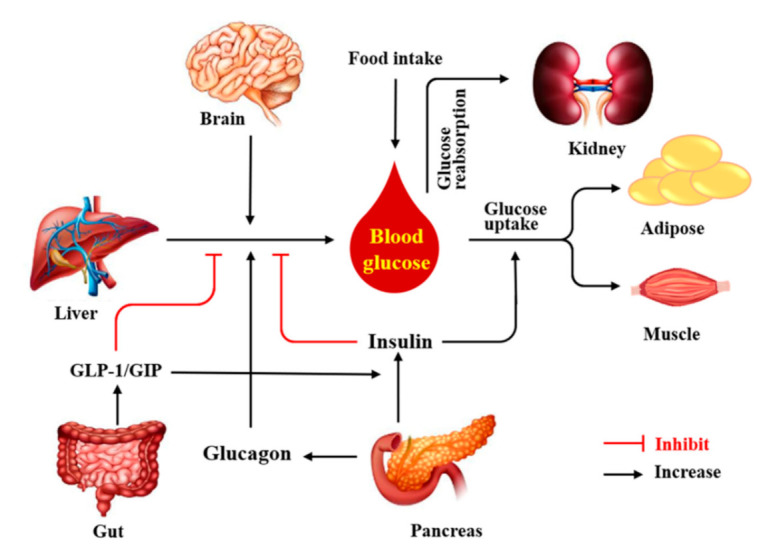
Coordination of various organs is required in the regulations of glucose homeostasis.

Diabetes is a chronic, metabolic disease characterized by elevated levels of blood glucose (or blood sugar), which leads over time to serious damage to the heart, blood vessels, eyes, kidneys and nerves according to World health Organization [1]. Globally, the number of diabetes cases is estimated at 463 million adults in 2019 and projected to increase to nearly 700 million diabetic individuals 25 years later, according to the International Diabetes Federation [2]. Diabetes remains one of the top ten causes of adult death, with an estimated 4 million deaths globally in 2017 [2]. At present, reasonable control of blood glucose and effective prevention and treatment of diabetic complications are the basic principles of diabetes treatment [3,4,5,6]. Glycemic homeostasis is regulated by complex mechanisms of endocrine signals derived primarily from the pancreas, liver, gut, skeletal muscle, adipose tissue, brain, and kidney (Figure 1) [7,8,9,10].

Food intake and hepatic glucose production increase the glucose levels. Hepatic glucose production is increased by glucagon, inhibited by insulin, and regulated by neural signals from the brain. Insulin reduces blood glucose by mainly promoting glucose uptake in muscle and adipose tissue. The kidney contributes to glucose homeostasis through regulating the processes of glucose reabsorption.

Cyclic adenosine monophosphate (cAMP), a vital intracellular second messenger, is synthesized from adenosine triphosphate (ATP) by adenylyl cyclase (Adcy) and degraded by phosphodiesterase (PDE). It exerts various physiological effects by activating protein kinase A (PKA), the classical cAMP downstream effector. The pivotal role of cAMP signaling in regulating physiological and pathological processes has been reflected by the fact that many drugs targeting cAMP are used for various diseases [11,12,13,14].

Substantial progress has been made in our understanding of the molecular mechanisms by which cAMP mediates the glucose homeostasis in multiple organs. For example, the activation of cAMP/PKA signaling pathway by glucagon in liver increases phosphorylation of cAMP-response element binding protein (CREB) and promotes the expression of key enzymes related to gluconeogenesis such as glucose-6-phosphatase (G6pase) and phosphoenolpyruvate carboxykinase (PEPCK) [15]. In the pancreatic islet, PDE4 inhibitors is able to improve glucose homeostasis by enhancing cAMP-mediated insulin secretion [16]. Indeed, the routine drugs for the clinical treatment of type 2 diabetes, such as glucagon-like peptide 1 (GLP-1) receptor agonists (lixisenatide, exenatide, exenatide, semaglutide, albiglutide, dulaglutide, and liraglutide) and dipeptidyl peptidase 4 inhibitors (sitagliptin, saxagliptin, alogliptin, and linagliptin) act via cAMP. Moreover, metformin inhibits hepatic gluconeogenesis by suppressing glucagon-induced cAMP production in hepatocytes [17].

Accumulated evidence has demonstrated that various food-derived bioactive ingredients perform excellent pharmacological roles for diabetes prevention and treatment via targeting cAMP signaling pathway. For example, genistein, a soy isoflavone, directly acts on pancreatic β cells and increases insulin secretion through the activation of cAMP/PKA signaling pathway [18]. Oral administration of curcumin for 10 weeks restrains hepatic glucose production by blocking cAMP/PKA signaling in the high-fat diet (HFD) fed ICR mice [19]. In this review, we focus on previously published literature about the physiological or pathological activities of cAMP in different organs related to diabetes, including pancreatic islets, liver, gut, skeletal muscle, adipose tissues, brain, and kidney, and then discuss the dietary bioactive ingredients which regulate glucose homeostasis by targeting cAMP. We hope that the conclusion of this review provides a new strategy for the prevention and treatment of diabetes.

## 2. What Is a cAMP?

cAMP, a form of adenosine monophosphate, is composed of adenine, ribose, and a phosphate group. In 1957, Berthet et al. found that cAMP is the second messenger of hormones in cells and plays a role in cell signal transduction when they studied the mechanism of adrenaline promoting glycogen decomposition [20]. As a second messenger for signal transduction at the cellular level, cAMP is used for intracellular signal transduction conveying the cAMP-dependent pathway in many different organisms. cAMP synthesis is increased when the Adcy is activated by extracellular signaling molecules (i.e., epinephrine, glucagon, serotonin) that bind to G-protein-coupled receptors (GPCR) and activate the G protein (Gαs). In contrast, the activation of G protein (Gαi) by the extracellular signaling molecules such as neuropeptide Y or prostaglandin E2 results in inhibition of Adcy and downregulation of cAMP level [21,22].

**Figure 2:**
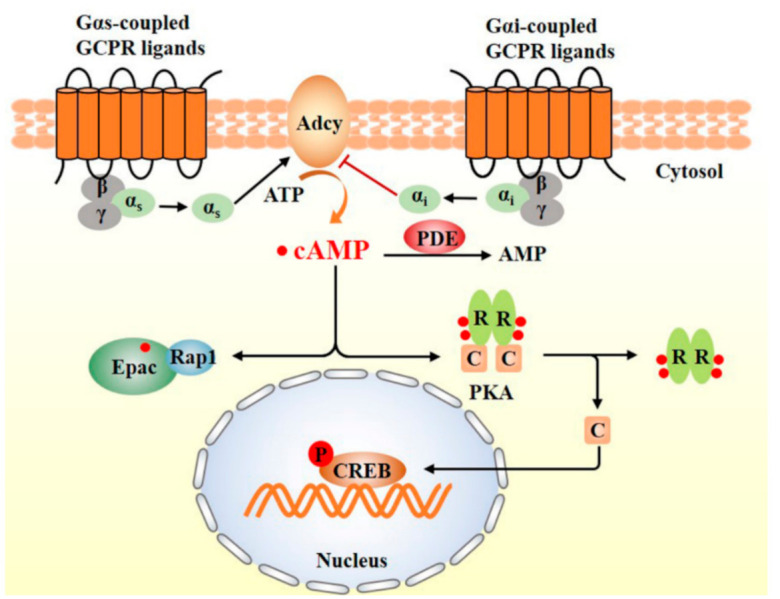
Schematic illustration of cAMP signaling pathway. Adcy: adenylyl cyclase; ATP: adenosine triphosphate; Epac: exchange proteins directly activated by cAMP; CREB: cAMP-response element binding protein; PDE: phosphodiesterase; PKA: protein kinase A.

The cAMP exerts its physiological effects by activating PKA or exchange proteins directly activated by cAMP (Epac), a PKA independent cAMP downstream effector. Briefly, cAMP binds to the regulatory subunit of PKA, resulting in the dissociation of PKA into regulatory and catalytic subunits, which may act on specific protein substrates and transfer phosphate to serine or threonine residues. For example, activated PKA acts on CREB, the famous downstream protein target of PKA, and further enhances or inhibits the expression of specific target genes, which in turn regulates the functions of a series of protein targets including ion channels, enzymes, and transcription factors (Figure 2) [23]. Epac was discovered by Kawasaki et al. in 1998 [24] and has known to play an important regulatory role in various important physiological and pathological processes, including endothelial cell angiogenesis [25], osteoclast function [26], and reducing oxidative stress [27].

## 3. Pancreas

### 3.1. cAMP Signaling Pathway

**Figure 3:**
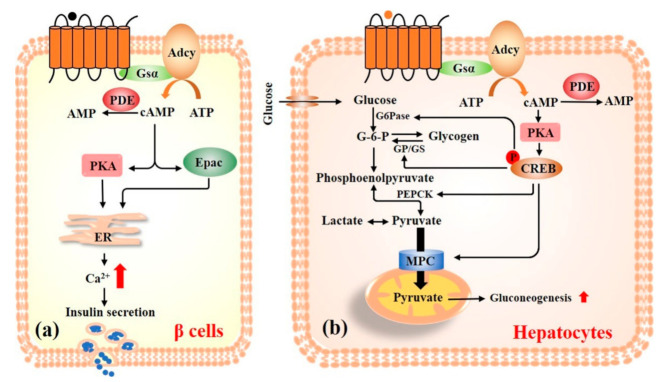
cAMP signaling pathway in the regulation of glucose homeostasis in pancreatic β cells (**a**) and hepatocytes (**b**). Adcy: adenylyl cyclase; ATP: adenosine triphosphate; Epac: exchange proteins directly activated by cAMP; CREB: cAMP-response element binding protein; G6pase: glucose-6-phosphatase; PDE: phosphodiesterase; PEPCK: phosphoenolpyruvate carboxykinase; PKA: protein kinase A; MPC: mitochondrial pyruvate carrier.

Insulin, secreted from the pancreatic islet β cells, facilitates the uptake and storage of glucose in response to elevated plasma glucose levels [28]. cAMP has a stimulatory effect on insulin secretion and acts as a positive coordinator in the glucose stimulated insulin secretion (Figure 3a). In the presence of glucose, an increase in cAMP production is induced, which drives the release of Ca^2+^ into the cell and increases store intracellular Ca^2+^ mobilization to stimulate insulin secretion. Hellman and Grill have found that the increase of cAMP concentration plays an important role in glucose-stimulated insulin secretion in β cells [29,30]. Elevated intracellular Ca^2+^ is considered as the final excitation mechanism for the insulin secretion from β cells. Later studies have verified that glucose stimulation increases the levels of cAMP, which is associated with the concentration of Ca^2+^ [31,32,33]. Therefore, cAMP is an amplifier of insulin secretion in β cells.

GLP-1, a gastrointestinal peptide, augments glucose-induced pancreatic β cell insulin secretion [34]. The intracellular GLP-1 signaling pathway involves cAMP, PKA, Epac, and K_ATP_ channels. Extracellular regulated protein kinases1/2 (ERK1/2) activation may be necessary for growth factors to drive cell proliferation [35]. Previous studies demonstrated that ERK1/2 phosphorylation is dependent on cAMP/PKA signaling activation in β cells and induces pancreatic β-cell proliferation [36]. The protection of pancreatic islet via targeting the cAMP signaling pathway is also an important strategy to promote glucose homeostasis.

cAMP also plays an important role in glucagon secretion in islet α cells. Glucose concentrations in the hypoglycemic range stimulate glucagon secretion by directly modulating the cAMP concentration in α cells independently of paracrine influences [37]. Glucagon release is positively modulated by the increased concentration of cytoplasmic Ca^2+^ elevated by cAMP in the guinea-pig α2-cells [38,39]. Meanwhile, cAMP triggers Ca^2+^ influx via voltage-dependent L- or N-type channels and increases glucagon secretion in α-cell [40,41]. Besides, adrenaline increases a large amount of cAMP that activates PKA and Epac and stimulates glucagon secretion in α-cell [42].

### 3.2. The Dietary Bioactive Ingredients Targeting Pancreas cAMP Signaling for Glucose Homeostasis

Over the past decades, dietary bioactive ingredients targeting cAMP in the islets for type 2 diabetes have received considerable interest due to their possible health and therapeutic benefits. In the following paragraphs, all of the selected nutraceuticals have a positive effect on insulin secretion or pancreatic protection, mainly including polyphenols (resveratrol, [6]-gingerol, and curcumin), flavonoids (genistein), polysaccharides (7WA and fucoidan), triterpenoid (oleanolic acid), and alkaloid (coixol) (Table 1).

Previous studies have reported that resveratrol has the ability to reduce blood glucose and improve insulin sensitivity by preventing the degradation of cAMP [43,44]. Additionally, a recent study reported that daily orally administration of [6]-gingerol for 28 days for db/db mice significantly increases glucose-stimulated insulin secretion and enhances glucose tolerance. Mechanistically, the anti-hyperglycemic activity of [6]-gingerol associated with the upregulation of cAMP, PKA, and CREB in the pancreatic islets, which are critical components of the GLP-1-mediated insulin secretion pathway [45]. Curcumin (1,7-bis-(4-hydroxy-3-methoxyphenyl)-hepta-1,6-diene-3,5-dione), a bioactive ingredient in ginger commonly used as coloring agent as well as food additive, increases insulin sensitivity by inhibiting PDE activity and increasing the level of cAMP in MIN6 and HP62 cells [43]. Both in vitro and in vivo, genistein (4,5,7-trihydroxyisoflavone), a flavonoid in legumes and some herbal medicines, has various biological actions including anti-diabetes. For example, genistein enhances pancreatic β cell proliferation and increases insulin secretion in INS1 cells and MIN6 cells, which is associated with increased intracellular cAMP levels and activated PKA [18,36,46]. In streptozotocin induced C57BL/6J diabetes mice, genistein improves hyperglycemia and glucose tolerance by the improvement of islet β cell proliferation and survival [36].

7WA, a type 2 arabinogalactan from green tea, enhances glucose-stimulated insulin secretion from RIN-5F cells, and its mechanism may involve the cAMP/PKA signaling pathway, as this progress is abrogated by Adcy and PKA inhibitor [47]. Moreover, Jiang et al. found that fucoidan, an extract of the seaweed *Fucus vesiculosus*, stimulates insulin secretion and provides pancreatic protection via the increase of intracellular cAMP levels in RIN-5F cells and Goto Kakizaki rats. Fucoidan-induced insulin secretion is significantly increased by PDE inhibitor, which decreases the degradation of cAMP, and is decreased by Adcy inhibitor, which decreases the generation of cAMP. This result indicated that fucoidan may stimulates insulin secretion via the cAMP signaling pathway [48].

Oleanolic acid (3-beta-hydroxyolean-12-en-28-oic acid), a pentacyclic triterpenoid compound with a widespread occurrence throughout the plant kingdom, increases insulin release in MIN6 cells and human islets through cAMP/Ca^2+^ signaling pathway [49]. Coixol, an alkaloid from *Scoparia dulcis*, significantly improves glucose tolerance and fasting blood glucose levels in streptozotocin-induced diabetic SD rats. This progress is significantly inhibited by the PKA inhibitor H-89 (50 μM) and Epac2 inhibitor MAY0132 (50 μM), indicating that coixol enhances insulin secretion by the cAMP signaling pathway [50]. Therefore, in the pancreas, the dietary bioactive ingredients targeting cAMP provide a new strategy for the promotion of insulin secretion and protection of pancreatic islets.

## 4. Liver

### 4.1. cAMP Signaling Pathway

cAMP signaling plays a crucial role in glycogenolysis and gluconeogenesis in the liver, a critical metabolic organ and a hub of energy metabolism (Figure 3b). For example, in the fasting state, the increased intracellular cAMP activates PKA and subsequently inhibits glycogen synthase and activates glycogen phosphorylase, rapidly increases glycogenolysis, and elevates blood glucose [51]. In addition to glycogenolysis, cAMP is also involved in the physiological processes of gluconeogenesis. Glucagon enhances glucose output from the liver by promoting the expression of PEPCK, G6pase, and peroxisome proliferator-activated receptor-γ coactivator-1α through cAMP/PKA/CREB pathway [52,53,54,55]. Moreover, one of the mechanisms by which metformin and other biguanides mitigate insulin resistance and diabetes is through inhibiting the glucagon-stimulated cAMP/PKA pathway [17,56]. Apart from glucagon, many other hormones regulate blood glucose through cAMP signaling pathway in the liver. For example, insulin inhibits gluconeogenesis in liver by suppressing cAMP/PKA/CREB pathway [57]. Leptin, like insulin, induces an intracellular signaling pathway in hepatocytes that increases the degradation of cAMP and antagonism of the actions of glucagon [58].

### 4.2. The Dietary Bioactive Ingredients Targeting Liver cAMP Signaling for Glucose Homeostasis

Products used in traditional Chinese medicines herbs and food ingredients are an important source in the search for safer drug alternatives, especially for diabetes, which is closely related to diet. In the following part, we will summarize the existing scientific evidence gleaned from a variety of in vitro/vivo studies that supports the beneficial impacts of PDE activator (curcumin, ginsenoside Rg5, phanginin A, astragaloside IV, and berberine) and Adcy inhibitor (ginsenoside Rb1, *Dendrobium officinale* polysaccharides, and epigallocatechin gallate) from dietary bioactive ingredients on the liver glucose homeostasis (Table 2).

PDE4B is an important target in the liver of curcumin, unlike its role in the pancreas. Orally administrated curcumin inhibits acetyl CoA production in HFD fed ICR mice by reducing mitochondrial fatty acid oxidation and inhibits hepatic glucose production through reducing cAMP accumulation and preserving PDE4B activity [19]. The predominant cAMP degradation in liver was thought to be under the effect of PDE4B [17]. Ginsenoside Rg5, a major saponin in steam-pretreated ginseng, inhibits gluconeogenesis and hepatic glucose production by reducing cAMP accumulation in hepatocytes [59]. Similarly, phanginin A, an isolated natural compound of the plant *Caesalpinia sappan*, inhibits gluconeogenesis by activating the activity of PDE4 and inhibiting the cAMP/PKA/CREB signaling pathway [15]. In addition, oral administration of astragaloside IV for 2 weeks inhibits adipose lipolysis and reduces hepatic glucose production via protein kinase B (Akt) dependent PDE3B expression, resultantly reducing adipose cAMP accumulation in HFD fed ICR mice [60]. Berberine, a quaternary ammonium protoberberine alkaloid with an isoquinoline scaffold isolated from medicinal herbs, promotes glucose uptake and inhibits gluconeogenesis via the activation of PDE activity and the reduction of cAMP level [61].

Unlike ginsenoside Rg5, Lou et al. found that ginsenoside Rb1 blocks cAMP signaling pathway by inhibiting Adcy activity and deactivating CREB through dephosphorylation, which may be helpful to inhibit the mitochondrial pyruvate carrier 1 and induce the reduction of liver glucose production [62]. *Dendrobium officinale* polysaccharide has many applications in medicine and food [65]. A recent study showed that *Dendrobium officinale* polysaccharides significantly inhibit the activity of Adcy and cAMP/PKA signaling pathways, resulting in the hepatic glycogen synthesis and hepatic glycogen degradation. Meanwhile, *Dendrobium officinale* polysaccharide decreases the expressions of G6pase and PEPCK, thereby inhibiting hepatic gluconeogenesis [63]. Epigallocatechin gallate, a major polyphenol from green tea, inhibits gluconeogenesis by antagonizing cAMP/PKA signaling pathway and inhibiting forkhead box O1 nuclear translocation [64].

## 5. Gut

### 5.1. cAMP Signaling Pathway

Incretins, like glucose-dependent insulinotropic polypeptide and GLP-1, are secreted by the enteroendocrine L cells and K cells after meal ingestion and are critical for maintaining glucose homeostasis by stimulating insulin secretion via cAMP signaling in a glucose-dependent manner [66]. Evidence has shown that luminal could significantly increase GLP-1 secretion in the colon and especially vascular infusion of acetate and butyrate via a cAMP-dependent way [67]. In addition, Vadder et al. reported that intestinal gluconeogenesis has beneficial effects on glucose and energy homeostasis. For example, intestinal gluconeogenesis gene expression is elevated under the effect of butyrate, a substrate of intestinal gluconeogenesis, through a cAMP-dependent mechanism [68].

### 5.2. The Dietary Bioactive Ingredients Targeting Gut cAMP Signaling for Glucose Homeostasis

In this section, we will review selected articles where 10 bioactive ingredients such as coffee polyphenols, geraniol, citronellal, delphinidin, resveratrol, *Polygonatum cyrtonema* polysaccharide, anthraquinone-glycoside, oleanolic acid, oleanolic acid derivative DKS26, and nonanoic acid were involved and highlight their effect and mechanism in the regulation of the secretion of GLP-1 via the cAMP-dependent pathway (Table 3).

Coffee, one of the most popular beverages due to its attractive flavor and potential health benefits, is rich in abundant dietary polyphenols. Administration of coffee polyphenol extract significantly increases GLP-1 secretion in NCI-H716 and C57BL/6J mice associated with increasing intracellular cAMP levels in a dose-dependent manner [69]. Geraniol, an acyclic mnonoterpene alcohol extracted from the ethereal oils of aromatic plants, and citronellal, a specialized metabolite of plants found in *Cymbopogon spp*., have a number of biological activities, such as antioxidant and antidiabetic properties [78]. Kim et al. found that geraniol- and citronellal-stimulated GLP-1 secretion in NCI-H716 cells are mediated by the activation of olfactory receptor 1G1, the activation of Adcy, increased intracellular cAMP levels, and extracellular calcium influx. In db/db mice, oral administration of geraniol improves glucose homeostasis by increasing plasma GLP-1 and insulin levels [70]. The inhibition of glucose transport in the gut could decrease the absorption of glucose, which could be beneficial for the management of diabetes. Some bioactive compounds inhibit glucose absorption in the gut via affecting cAMP signaling. For example, delphinidin, an anthocyanidin found in pigmented fruits and vegetables with anti-inflammatory, anti-angiogenic, and anti-oxidant properties, inhibits glucose absorption in both RF/J mice jejunum and a human enterocytic cell line (HT-29, Caco-2, and NCM460) in a free fatty acid receptor 1-dependent manner and affects the function of sodium-glucose cotransporter 1. The intracellular signaling is involved in cAMP increase and Ca^2+^ influx [71]. Resveratrol influences the intestinal transport of glucose, alanine in weaned piglet porcine jejunum, and ileum, at least partly, by increasing levels of cAMP [72].

*Polygonatum cyrtonema* Hua Polysaccharide, the major component of *P. cyrtonema* belonging to an edible plant *Liliaceae* family, promotes GLP-1 secretion in SD rats, C57BL/6J mice, and NCI-H716 cells. GLP-1 secretion is significantly suppressed by the inhibitor of sweet taste receptor and Adcy inhibitor, suggesting that this progress is regulated by the sweet taste receptor-mediated cAMP signaling [73]. Anthraquinone-glycoside, purified from rhubarb, decreases fasting blood glucose and increases GLP-1 concentrations in SD rats associated with the regulation of the gut microbiota, the activation of the GLP-1/cAMP pathway, and the amelioration of insulin resistance [74].

Oleanolic acid (3beta-hydroxyolean-12-en-28-oic acid), a common pentacyclic triterpenoid mainly found in olive oil as well as several plant species, has several promising pharmacological activities including anti-inflammatory, antioxidant, or anti-cancer activities [79]. Oleanolic acid activates Gαs-coupled the bile acid G protein-coupled receptor 5 and promotes both GLP-1 and peptide YY (PYY) secretion via a PKA-independent, cAMP-dependent mechanism involving Epac/phospholipase C/Ca^2+^ pathway [75]. DKS26, a novel compound derived from oleanolic acid, significantly augments glucose consumption in human hepatic HepG2 cells and enhance GLP-1 release and expression in NCI-H716 L cells mediated by the activation of the cAMP/PKA signaling pathway [76]. Besides, nonanoic acid, a medium-chain fatty acid found in a wide variety of foods, increases GLP-1 and PYY secretion in NCI-H716 cells. Oral administration of nonanoic acid to SD rats results in about three times fold increase in GLP-1 levels and reductions in blood glucose levels compared with the control group. The primary mechanism is associated with olfactory receptor 51E1/cAMP/PKA signaling [77].

## 6. Skeletal Muscle

### 6.1. cAMP Signaling Pathway

Glucose uptake in skeletal muscle is the rate-limiting step for whole-body glucose metabolism [80]. Activation of the phosphoinositol 3-kinase/Akt pathway results in translocation of insulin-sensitive glucose transporter 4 (GLUT4) to the plasma membrane and an increase of glucose transport into skeletal muscle is the traditional insulin pathway [81]. A novel physiological system that increases glucose uptake in skeletal muscle involves activation of cAMP signaling pathway and independent of insulin [82]. The pathway involves activation of β2-adrenoceptors that increase cAMP levels and activate PKA, which phosphorylates mammalian target of rapamycin complex 2 at S2481. The active phosphorylates mammalian target of rapamycin complex 2 causes translocation of GLUT4 to the plasma membrane and glucose uptake without the involvement of insulin/Akt signaling [82]. Besides, Mukaida et al. also reported that BRL37344, a dual β_2_-/β_3_-adrenoceptor agonist, improves glucose tolerance and increases glucose uptake into skeletal muscle in vivo and ex vivo through a β_2_-adrenoceptor-mediated mechanism independently of Akt and partly associated with cAMP [83].

Skeletal muscle is the major site for glycogen storage, accounting for nearly 80% of the total glycogen [84]. Unlike liver, skeletal muscles’ glycogen is unable to release glucose into the blood to maintain blood glucose homeostasis, as skeletal muscles lack G6Pase for glycogenolysis [85]. Adrenaline or norepinephrine stimulates skeletal muscle glycogen depletion by activation of the β-adrenergic receptor/cAMP/PKA signaling pathway and activation of glycogen phosphorylase and inactivation of glycogen synthase in exercise stated to meet the energy demands [84,86]. Many studies have shown that regulation of cAMP signaling pathway in skeletal muscle can serve as an alternative pathway for glucose disposal and glycemic control. 

### 6.2. The Dietary Bioactive Ingredients Targeting Skeletal Muscle cAMP Signaling for Glucose Homeostasis

Promotion of glucose uptake and inhibition of insulin resistance are the main strategy by targeting skeletal muscle cAMP for glucose homeostasis in skeletal muscle. Four compounds were collected in the literature: daidzein, cyanidin-3-O-β-glucoside, α-cedrene, and [6]-gingerol, which are abundant in various plants and show acceleration of glucose uptake into skeletal muscle cells through the promotion of GLUT4 translocation to plasma membrane. Resveratrol, genistein, and oleic acid ameliorates insulin resistance in skeletal muscle cells through the increase of the level of cAMP and the inhibition the activity of PDE in skeletal muscle cells (Table 4).

Both in vitro and in vivo, daidzein, a natural isoflavone with estrogen-like activity mainly from fermented soybean, and cyanidin-3-O-β-glucoside, widely exists in deep-colored fruits and vegetables, significantly promote glucose uptake into skeletal muscle cells through the promotion of GLUT4 translocation to plasma membrane. This progress is through inhibiting the activity of PDE and increasing the level of intracellular cAMP in L6 myocytes and KK-Ay mice [87,88]. Similarly, α-cedrene, the ligand of mouse olfactory receptor 23, stimulates glucose uptake by stimulating the GLUT4 translocation to the cell membrane in the C2C12 myotubes via mouse olfactory receptor 23/cAMP/PKA signaling pathway [89]. In vivo, Samad et al. reported that [6]-gingerol, a major component of zingiber officinale, improves hyperglycemia by stimulating GLUT4 transporters translocation to cytomembrane in skeletal muscle [45].

In vitro, resveratrol, a polyphenol in red wine present in grapes, mulberries, peanuts, rhubarb, and in several other plants, inhibits cAMP degrading PDE activity and increases cAMP levels in C2C12 myotubes. Administration of resveratrol to C57BL/6J mice increases the levels of cAMP in skeletal muscle. It suggests that it has the potential of antidiabetogenic properties [90]. Genistein promotes glucose uptake by inhibiting PDE activity and increasing the level of intracellular cAMP level in L6 myotubes [91]. Oleic acid, monounsaturated long chain fatty acids, modulates the rates of fatty acid oxidation, ameliorates insulin resistance, and decreases inflammation in skeletal muscle cells and Wistar rats via stimulating the cAMP/PKA pathway, respectively [92,93]. Taken together, these genetic and pharmacological studies have shown that appropriately regulated cAMP/PKA activity is essential for skeletal muscle glucose uptake and systemic glucose homeostasis.

Some compounds such as resveratrol also have antioxidant, anti-inflammatory, and immunomodulatory effects [94]. Therefore, in addition to their regulatory effect on cAMP level, their diverse modulating activities (antioxidant, anti-inflammatory, immunomodulatory on different tissues and cells) may be responsible for the anti-diabetes effect; however, this needs to be confirmed via further investigation.

## 7. Adipose Tissue

### 7.1. cAMP Signaling Pathway

The cAMP/PKA signaling pathway in adipose mainly mediates glucose uptake and lipolysis in both white adipose tissue and brown adipose tissue. Although the uptake of glucose in adipose accounts for only 10%, it serves as a crucial integrator of energy balance and glucose homeostasis [95]. Similarly, with skeletal muscles, the main pathway to promote glucose uptake is by facilitating the translocation of GLUT, the translocation of which is also mainly involved in two different pathways, including the insulin-dependent stimulated signaling pathway and the insulin-independent cAMP signaling pathway. Olsen et al. reported that β_3_-adrenoceptors stimulate glucose uptake in brown adipose tissue via a signaling pathway that is comprised of two different parts: one part dependent on cAMP-mediated increases in GLUT1 transcription and de novo synthesis of GLUT1 and another part dependent on mammalian target of rapamycin 2 stimulated translocation of newly synthesized GLUT1 to the plasma membrane, leading to increased glucose uptake [96].

Lipolysis, which are strongly associated with obesity development, insulin resistance, and type 2 diabetes, starts with the hormone binding to GPCR on the adipocytic membrane, and then activates Adcy, promotes the synthesis of cAMP, and activates PKA. Activation of adipose triglyceride lipase, hormone-responsive lipase, and lipid droplet-associated protein perilipin A is phosphorylated by PKA, resulting in the release of glycerol and free fatty acids (FFAs) [97,98,99]. Lipolysis increases the levels of FFAs into the circulation and contributes to insulin resistance and diabetes. The possibility might be considered that FFAs themselves participate in the various organ defects which precede type 2 diabetes. As cAMP signaling is located at the upstream of lipolysis cascades, the regulation of cAMP is a key determinant in the control of the downstream lipolysis process [60]. The lipolysis is inhibited by suppressing cAMP production and PKA activation in adipocytes under the action of metformin for diabetes [100,101].

### 7.2. The Dietary Bioactive Ingredients Targeting Adipose Tissue cAMP Signaling for Glucose Homeostasis

In adipose tissues, some bioactive ingredients improve glucose homeostasis by promoting GLUT4 translocation (α-cedrene and pachymic acid) and inhibiting lipolysis (curcumin and ginsenoside Rg5) (Table 5). In addition, some components (piperonal and filbertone) targeting adipose cAMP signaling that have great potential to maintain glucose homeostasis are also introduced in this part.

α-Cedrene, a member of the class of organic compounds known as cedrane and iso-cedrane sesquiterpenoids, stimulates glucose uptake by stimulated the GLUT4 translocation to the cytomembrane in the C2C12 myotubes and 3T3-L1 adipocytes via mouse olfactory receptor 23/cAMP/PKA pathway [89]. Pachymic acid, one of the major chemical components of *Poria cocos*, stimulates glucose uptake via increasing the expression of GLUT4 and enhancing the redistribution of GLUT4 from intracellular vesicles to the plasma membrane of 3T3-L1 adipocytes. It also promotes lipid storage by inhibiting the formation of cAMP in adipocytes and thereby reduces lipolysis [102].

In vivo studies show that oral administration of curcumin decreases adipose lipolysis by attenuating endoplasmic reticulum stress through the cAMP/PKA pathway, reduces FFA influx into the liver by blocking FFA trafficking, and thereby improves insulin sensitivity to inhibit hepatic glucose production [103]. Treatment of ginsenoside Rg5 inhibits lipolysis and insulin resistance in HFD fed ICR mice through enhancing the expression of PDE3B and then reducing the accumulation of cAMP [104].

Piperonal, an aromatic compound found in vanilla and filbertone, the main flavor compound in hazelnuts, increases the level of cAMP in adipose tissue, decreases the body weight and plasma lipid profiles, and improves glucose tolerance in HFD-induced obese mice [105,106,107]. Obesity is well-known to contribute to the progression and development of diabetes. Therefore, it is interesting to confirm the anti-diabetes effects of piperonal and filbertone, and investigate the underling mechanism.

In the adipose tissue, it appears that both increase and decrease of cAMP can enhance glucose uptake. cAMP decrease seems to be possibly responsible for insulin resistance. This mixed result underscores a certain difficulty in schematizing the effect of all these compounds based on their activity on intracellular cAMP and highlights the limitations of adopting in vitro models. Further in vivo study is needed to parse the exact physiological roles of cAMP in glucose homeostasis.

Many of the antidiabetogenic effects of these compounds reported are tested in vitro (for example in cultivated adipocytes). As the greatest part of phyto-derived molecules undergoes the tight control of ATP binding cassette transporter and hepatic enzymatic transformation [108], which dramatically decrease their bioavailability and effectiveness, the in vivo/human confirmatory studies is therefore urgently needed.

## 8. Brain

The brain plays a key role in regulating the energy balance and glucose homeostasis by cooperating with liver, skeletal muscle, adipose, pancreatic islets, gut, etc. [109,110,111]. Peripheral signals, including hormones, metabolites, and neural afferent signals, are received and processed by the brain, triggering appropriate behavioral and metabolic responses to maintain energy and glucose homeostasis [112]. The cAMP signaling pathway in multiple cerebral regions mediates the physiological effects of many hormones and neurotransmitters. For example, in hypothalamic neurons, cAMP signaling is modulated by several hormones such as insulin, leptin, glucagon, and GLP-1. As mentioned above, leptin regulates glucose homeostasis by affecting the hypothalamus neurons [113]. Hypothalamic glucagon signaling inhibits hepatic glucose production via the activation of hypothalamic cAMP/PKA pathway. Inhibition of PKA activity in the mediobasal hypothalamus abolishes the suppressive effect of glucagon on hepatic glucose production and leads to increased i.v. glucagon injection-induced hepatic glucose production. Conversely, hepatic glucose production is inhibited at the effect with the activation of PKA in the mediobasal hypothalamus or glucagon injection to the hypothalamus [112,114]. In summary, cAMP/PKA signaling pathway manipulations in the brain, most likely in hypothalamic neurons, has profound effects to improve glycemic control in diabetes.

Zhang et al. showed that resveratrol promotes cellular glucose utilization by increasing the content of intracellular cAMP as well as Ca^2+^ in cortical neurons [115]. To date, there have been fewer studies targeting the brain to investigate the dietary bioactive ingredients that target cAMP signaling to control blood glucose. On the one hand, it is suggested that there is still a great research potential worth developing here, and on the other hand, it is also precisely the difficult point of research because most of the compounds have difficulty penetrating directly through the blood–brain barrier to see a direct effect. Although the existing studies suggest that there are few substances in food that play a role in regulating blood glucose balance by affecting cAMP in the brain, in the future, the researcher may focus on whether those compounds have the function of regulating blood glucose balance owing to its effect on cAMP potential and the physiological characteristics of the brain, as well as the properties of compounds.

## 9. Kidney

The kidney contributes to glucose homeostasis through processes of gluconeogenesis, glucose filtration, glucose reabsorption, and glucose consumption via sodium-glucose transporters (SGLTs) and glucose transporters [116]. In the fasted state of healthy individuals, 20–25% of glucose released into the circulation derives from the kidneys and the remaining 75–80% derives from the liver with glycogenolysis and gluconeogenesis [117,118]. Glucose reabsorption in the proximal tubule (via SGLT1 and SGLT2) has emerged as an important contributor to the development of diabetes. Inhibition of SGLT2 is a viable treatment option for patients with type 2 diabetes and delays the development of diabetic kidney disease. SGLT2 inhibitors, such as dapagliflozin, ertugliflozin, empagliflozin, and canagliflozin, have been approved by the FDA for use as a treatment for diabetes since 2013, which works by preventing the kidneys from reabsorbing glucose back into the blood. A recent study reported that elevated glucose concentration from 5 mM to 17.5 mM in culture media decreases trafficking of SGLT2 to the plasma membrane in pig kidney cells (LLC-PK_1_ cells) via reducing intracellular cAMP [119]. β-adrenergic agonists increase cAMP level and further stimulate SGLT1-mediated glucose uptake, which was inhibited by PKA inhibitor H-89 in sheep and rats [120,121]. Forementioned studies show that inhibition of SGLT in the kidneys to reduce blood glucose levels by modulating the cAMP signaling was an important strategy for the treatment of diabetes.

Sophora flavanone G, a prenylated flavanone from the roots of *Sophora flavescens Aiton*, is characterized as an SGLT inhibitor [122]. Another study shows that sophora flavanone G plays an anti-cancer role in non-small-cell lung cancer cells by acting on cAMP signaling pathway [123]. However, there is no clear study on whether sophora flavanone G can inhibit SGLT through the cAMP signal. Many studies have suggested that numerous bioactive compounds, such as naringenin, phlorizin, and other compounds, inhibit the process of glucose reabsorption in kidneys by inhibiting the activity of SGLT [124,125,126]. cAMP signal plays an important role in inhibiting glucose reabsorption by inhibiting SGLT. Whether the specific mechanism of those bioactive compounds affect cAMP signaling will also be an interesting and worthy research direction.

## 10. Conclusions

cAMP signaling in different organs has essential effects on whole-body glucose homeostasis and regulates glucose metabolism at multiple levels. A large amount of evidence has demonstrated that a diversity of dietary bioactive ingredients activate or inhibit cAMP signaling, and interfere with related pathophysiological processes, mainly including the promotion of insulin, GLP-1, PYY secretion, and glucose uptake, the inhibition of gluconeogenesis, lipolysis, glucose absorption, and hepatic glucose production, and the protection of the pancreas. The findings, mainly from in vitro studies, indicate that certain bioactive compounds, such as curcumin, resveratrol, and genistein, play a major role in modulating cAMP signaling and regulating diabetes; nevertheless, future confirmatory in vivo studies are needed. Further exploration of dietary bioactive ingredients targeting the cAMP signaling will provide new opportunities for diabetes treatment, and for the improvement of quality of life.

## Figures and Tables

**Table 1:** The dietary bioactive ingredients targeting pancreas cAMP signaling for glucose homeostasis.

Compounds	Main Source of Food	Concentration of cAMP	Main Effect	Mechanism of Action	Model	Refs.
Resveratrol	Peanut; grape (red wine); mulberry	• ↑ cAMP	• ↓ Blood glucose • ↑ Insulin sensitivity	• Inhibition of PDE activity	• MIN6 cells • HP62 cells	[43]
		• ↑ cAMP	• ↑ Insulin secretion	• Activation of cAMP/Epac1signaling pathway	• C57BL/6N mice	[44]
[6]-Gingerol	Ginger	• ↑ cAMP	• ↑ Insulin secretion	• Activation of cAMP/PKA signaling pathway	• db/db mice	[45]
Curcumin	Ginger	• ↑ cAMP	• ↑ Insulin sensitivity	• Inhibition of PDE activity	• MIN6 cells • HP62 cells	[43]
Genistein	Soy; locust horn	• ↑ cAMP	• ↑ Pancreatic β-cell proliferation	• Activation of cAMP/PKA MEK/ERK signaling • Phosphorylation of ERK1/2	• INS-1 cells • PANC1s cells	[36]
		• ↑ cAMP	• ↑ Insulin secretion	• Activation of the Adcy/cAMP/PKA signaling pathway	• INS-1 cells • MIN6 cells	[18]
		• ↑ cAMP	• ↑ Insulin secretion	• Accumulation of cAMP and Ca^2+^	• MIN6 cells	[46]
		• ↑ cAMP	• ↓ Hyperglycemia • ↑ Glucose tolerance	• Improvement of islet β-cell proliferation, survival, and mass	• C57BL/6J mice	[36]
7WA	Green tea	• ↑ cAMP	• ↑ Insulin secretion	• Activation of cAMP/PKA signaling pathway	• RIN-5F cells	[47]
Fucoidan	Seaweed Fucus vesiculosus	• ↑ cAMP	• ↑ Insulin secretion • ↓ Hyperglycemia • ↑ Pancreatic β-cell proliferation	• Activation of cAMP/PKA and PI3K/Akt signaling pathway	• INS-1E cells • Human islets	[48]
Oleanolic acid	Jujube; papaya	• ↑ cAMP	• ↑ Insulin secretion	• Activation Gs/cAMP/Ca^2+^ signaling pathway	• MIN6 cells	[49]
Coixol	Coix chinensis	• ↑ cAMP	• ↑ Insulin secretion	• Activation of cAMP/PKA signaling pathway	• MIN6 cells	[50]

Abbreviation: Adcy: adenylyl cyclase; Akt: protein kinase B; cAMP: cyclic adenosine monophosphate; CREB: cAMP-response element binding protein; ERK: extracellular regulated protein kinases; MEK: mitogen-activated protein kinase kinase; PDE: phosphodiesterase; PI3K: phosphatidylinositide 3-kinases; PKA: protein kinase A; ↑: Increase or promote; ↓: Decrease or inhibit.

**Table 2:** The dietary bioactive ingredients targeting liver cAMP signaling for glucose homeostasis.

Compounds	Main Source of Food	Concentration of cAMP	Main Effect	Mechanism of Action	Model	Refs.
Curcumin	Ginger	• ↓ cAMP	• ↓ Hepatic glucose production	• Preservation of PDE4B activity • Inhibition of acetyl CoA production	• ICR mice • Primary hepatocytes • HepG2 cells	[19]
Ginsenoside Rg5	Ginseng	• ↓ cAMP	• ↓ Hepatic gluconeogenesis	• Preservation of PDE4B activity	• C57BL/6J mice • Primary hepatocytes	[59]
Phanginin A	*Caesalpinia sappan*	• ↓ cAMP	• ↓ Hepatic gluconeogenesis	• Activation of PDE4 activity • Inhibition of cAMP/PKA/CREB signaling pathway	• C57BL/6J mice • Primary hepatocytes	[15]
Astragaloside IV	Astragalus membranaceus	• ↓ cAMP	• ↓ Hepatic lipid deposition • ↓ Hepatic glucose production	• Preservation of PDE3B activity	• ICR mice	[60]
Berberine	Cortex phellodendri; coptis chinensis	• ↓ cAMP	• ↓ Hepatic gluconeogenesis	• Activation of PDE activity	• ob/ob mice	[61]
		• ↓ cAMP	• ↓ Hepatic gluconeogenesis	• Inhibition of glucagon signaling	• Primary hepatocytes	
Ginsenoside Rb1	Ginseng	• ↓ cAMP	• ↓ Hepatic gluconeogenesis	• Inhibition of Adcy activity • Inactivation of CREB	• C57BL/6J mice • Primary hepatocytes	[62]
*Dendrobium officinale* polysaccharide	*Dendrobium officinale*	• ↓ cAMP	• ↑ Hepatic glycogen synthesis • ↓ Hepatic glycogen degradation • ↓ Hepatic gluconeogenesis	• Inactivation of Adcy activity • Reduction the expression of PKA	• C57BL/6J mice	[63]
Epigallocatechin gallate	Green tea	ND	• ↓ Hepatic glucose production	• Inhibition of cAMP/PKA/CREB signaling pathway	• Primary hepatocytes	[64]

Abbreviation: Adcy: adenylyl cyclase; cAMP: cyclic adenosine monophosphate; CREB: cAMP-response element binding protein; ND: not detection; PDE: phosphodiesterase; PEPCK: phosphoenolpyruvate carboxykinase; PKA: protein kinase A; ↑: Increase or promote; ↓: Decrease or inhibit.

**Table 3:** The dietary bioactive ingredients targeting gut cAMP signaling for glucose homeostasis.

Compounds	Main Source of Food	Concentration of cAMP	Main Effect	Mechanism of Action	Model	Refs.
Coffee polyphenols	Coffee, goji berries	• ↑ cAMP	• ↑ GLP-1 secretion	• Activation of cAMP-dependent pathway	• NCI-H716 cells	[69]
Geraniol	Geranium; lemon	• ↑ cAMP	• ↑ GLP-1 secretion	• Activation of OR1G1 /cAMP/PKA signaling pathway	• NCI-H716 cells	[70]
Citronellal	Kaffir lime leaves	• ↑ cAMP	• ↑ GLP-1 secretion	• Activation of OR1G1/cAMP/PKA signaling pathway	• NCI-H716 cells	[70]
Delphinidin	Bilberry; Maqui berry	• ↑ cAMP	• ↓ Glucose uptake	• Activation of FFA1/GPR40/cAMP signaling pathway	• RF/J mice • HT-29 cells • Caco-2 cells • NCM460 cells	[71]
Resveratrol	Peanut; grape (red wine); mulberry	• ↑ cAMP	• ↓ Glucose and alanine transport	• Inhibition of PDE activity	• Porcine jejunum and ileum	[72]
*Polygonatum cyrtonema* polysaccharide	*Polygonatum cyrtonema*	• ↑ cAMP	• ↑ GLP-1 secretion	• Activation of the T1R2/T1R3/cAMP signaling pathway	• NCI-H716 cells	[73]
Anthraquinone-glycoside	Rhubarb	• ↑ cAMP	• ↑ GLP-1 secretion	• Activation of cAMP signaling pathway	• SD rats	[74]
Oleanolic acid	Jujube; Papaya	• ↑ cAMP	• ↑ GLP-1 and PYY secretion	• Activation of TGR5/cAMP/Epac/Ca^2+^ signaling pathway	• STC-1 cells	[75]
Oleanolic acid derivative DKS26	Hawthorn	• ↑ cAMP	• ↑ GLP-1 secretion	• Activation of the cAMP/PKA signaling pathway	• db/db mice • NCI-H716 cells	[76]
Nonanoic Acid	Royal jelly	• ↑ cAMP	• ↑ GLP-1 and PYY secretion	• Activation of OR51E1/cAMP and p-ERK signaling pathway	• SD rats • NCI-H716 cells	[77]
Butyrate	Cheese	• ↑ cAMP	• ↑ Insulin sensitivity	• Activation of cAMP dependent pathway	• SD rats • Caco-2 cells	[68]

Abbreviation: cAMP: cyclic adenosine monophosphate; Epac: Exchange proteins directly activated by cAMP; ERK: extracellular regulated protein kinases; GLP-1: Glucagon-like peptide-1; OR1G1: olfactory receptor 1G1; OR51E1: olfactory receptor 51E1; PDE: phosphodiesterase; PKA: protein kinase A; PYY: Peptide YY; TGR5: the bile acid G protein-coupled receptor; SD: Sprague-Dawley; ↑: Increase or promote; ↓: Decrease or inhibit.

**Table 4:** The dietary bioactive ingredients targeting skeletal muscle cAMP signaling for glucose homeostasis.

Compounds	Main Source of Food	Concentration of cAMP	Main Effect	Mechanism	Model	Refs.
Daidzein	Soy; celery	• ↑ cAMP	• ↑ Glucose uptake	• Inhibition of PDE4 activity • Improved the AMPK phosphorylation	• L6 cells • db/db mice	[87]
Cyanidin-3-O-β-glucoside	Black soybean; blueberry	• ↑ cAMP	• ↑ Exercise performance	• Inhibition of PDE activity	• ICR mice • C2C12 cells	[88]
α-Cedrene	Cedar wood oil	• ↑ cAMP	• ↑ Glucose uptake	• Activation of OR23/cAMP/PKA signaling pathway	• C2C12 cells	[89]
[6]-Gingerol	Ginger	• ↑ cAMP	• ↑ Glycogen deposition	• Activation of cAMP/PKA/CREB signaling pathway	• db/db mice	[45]
Resveratrol	Grape; mulberry	• ↑ cAMP	• ↑ Glucose uptake	• Inhibition of PDE4 activity	• C2C12 cells	[19,90]
Genistein	Soy	• ↑ cAMP	• ↑ Glucose uptake	• Inhibition of PDE activity	• L6 cells	[91]
Oleic acid	Walnuts; nuts; almonds	• ↑ cAMP	• ↑ Oxidation of fatty acids	• Activation of Sirtin1-PGC1α complex	• C57BL/6 mice	[64,92]
		• ↑ cAMP	• ↑ Insulin sensitivity	/	• Wistar rats	[93]

Abbreviation: AMPK: adenosine 5’-monophosphate-activated protein kinase; cAMP: cyclic adenosine monophosphate; CREB: cAMP-response element binding protein; mOR23: mouse olfactory receptor 23; PDE: phosphodiesterase; PGC-1α: peroxisome proliferator-activated receptor- γ coactivator; PKA: protein kinase A; ↑: Increase or promote; ↓: Decrease or inhibit.

**Table 5:** The dietary bioactive ingredients targeting adipose tissue cAMP signaling for glucose homeostasis.

Compounds	Main Source of Food	Concentration of cAMP	Main Effect	Mechanism of Action	Model	Refs.
α-Cedrene	Cedar wood oil	• ↑ cAMP	• ↑ Glucose uptake	• Stimulation of mOR23/cAMP/PKA signaling pathway	• 3T3-L1 adipocytes	[89]
Pachymic acid	Poria cocos	• ↓ cAMP	• ↑ Glucose uptake • ↓ Lipolysis	• Stimulation of GLUT4 expression and redistribution	• 3T3-L1 adipocytes	[102]
Curcumin	Ginger	• ↓ cAMP	• ↓ Lipolysis	• Reduction of ER stress	• C57BL/6 mice • 3T3-L1 adipocytes	[103]
Ginsenoside Rg5	Ginseng	• ↓ cAMP	• ↓ Lipolysis • ↑ Insulin resistance	• Preservation of PDE3B activity	• 3T3-L1 adipocytes	[104]

Abbreviation: cAMP: cyclic adenosine monophosphate; ER: endoplasmic reticulum; GLUT4: glucose transporter 4; mOR23: mouse olfactory receptor 23; PDE: phosphodiesterase; PKA: protein kinase A; ↑: Increase or promote; ↓: Decrease or inhibit.

## Data Availability

Not applicable.
